# Blood glucose control with different treatment regimens in type 2 diabetes patients hospitalized with COVID-19 infection: A retrospective study

**DOI:** 10.1097/MD.0000000000032650

**Published:** 2023-01-20

**Authors:** Elena Chertok Shacham, Nimra Maman, Avraham Ishay

**Affiliations:** a Endocrinology Unit, Haemek Medical Center, Afula, Israel; b Statistic Department, Haemek Medical Center, Afula, Israel; c Faculty of Medicine, Technion – Israel Institute of Technology, Haifa, Israel.

**Keywords:** COVID-19, diabetes therapy in the hospital, type 2 diabetes

## Abstract

Coronavirus disease (COVID-19) is closely associated with hyperglycemia and a worse prognosis in patients with a previous diagnosis of type 2 diabetes mellitus. A few studies investigated the effects of diabetes treatment regimens in these patients during hospitalization. Here, we evaluate the impact of insulin and non-insulin therapy on glucose control in patients with type 2 diabetes admitted with COVID-19.

This is a retrospective study including 359 COVID-19 patients with type 2 diabetes. Patients were divided into 2 groups according to diabetes treatment during hospitalization. The first group included patients treated with insulin only, and the second group patients treated with other antidiabetic agents with or without insulin. Average blood glucose was higher in the insulin-only treatment group (201 ± 66 mg/dL vs 180 ± 71 mg/dL, *P* = .004), even after excluding mechanically ventilated patients (192 ± 69 vs 169 ± 59 mg/dL, *P* = .003). In patients with moderate severity of COVID-19, average blood glucose was also significantly higher in the insulin-only treated group (197 ± 76 vs 168 ± 51 mg/dL, *P* = .001). Most patients (80%) in the combination treatment group received metformin. Moderately affected COVID-19 patients with type 2 diabetes could safely be treated with antihyperglycemic medications with or without insulin.

**Key points**

Patients with type 2 diabetes could be treated with oral antihyperglycemic medicine with or without insulin during hospitalization according to their general status. Type 2 diabetes patients with severe coronavirus disease 2019 should be treated with basal-bolus insulin protocol. Further studies are required to evaluate the role of sodium-glucose transport protein inhibitors during hospitalization.

## 1. Introduction

Severe acute respiratory syndrome coronavirus 2 infection was found to be closely associated with hyperglycemia in patients without diabetes and with worse outcomes in patients previously diagnosed with type 2 diabetes mellitus.^[1–3]^ Hyperglycemia per se is proposed to be a disease modifier, among other mechanisms, by impairing the function of innate and adaptive immune systems and perpetuating inflammatory responses.^[4]^

In patients with diabetes mellitus, the upregulation of angiotensin-converting enzyme 2 expression in cardiomyocytes could increase the susceptibility to coronavirus disease 2019 (COVID-19) infection and worsen prognosis.^[5]^ The prevalence of diabetes in patients admitted to hospitals with COVID-19 varies between 8.2 and 35% and is related to adverse outcomes.^[6,7]^ Patients with type 2 diabetes have higher rates in an intensive care unit when compared with patients without diabetes (37.0% vs 26.7%, respectively; *P* = .028).^[8]^ In a recent retrospective study among 1122 COVID-19 patients hospitalized, the mortality rate was 28.8% in patients with diabetes and/or uncontrolled hyperglycemia compared with 6.2% in patients with normoglycemia.^[9]^ In another retrospective study of >7000 COVID-19 patients, the authors noticed that in COVID-19 patients with preexisting diabetes, well-controlled blood glucose was correlated with reduced risk of all-cause mortality and less frequent complications, including adult respiratory distress syndrome, acute kidney injury, and septic shock.^[10]^ Hyperglycemia can trigger endothelial damage, and over-inflammation via an increase in the expression of cytokines, including interleukin-6 and tumor necrosis factor-α in severely ill COVID-19 patients.^[11]^ Recent studies showed that patients with uncontrolled type 2 diabetes had reduced titers of COVID-19 neutralizing antibodies after severe acute respiratory syndrome coronavirus 2 vaccination and higher rates of breakthrough COVID-19 infection one year after vaccination.^[12,13]^

Insulin is well-established as the most appropriate pharmacologic agent for effectively controlling glycemia in hospitals, particularly in critically ill patients.^[14]^ Insulin regimens that include basal, prandial, and corrective doses result in better glucose control and fewer complications during hospitalizations.^[15]^ In the last decade, new classes of antidiabetic drugs are increasingly used to improve diabetes control.^[16–18]^ Furthermore, glucagon-like peptide-1 receptor agonists (GLP-1 RA) exhibits anti-inflammatory actions in animals with experimental inflammation and reduces biomarkers of systemic inflammation in patients with obesity and type 2 diabetes.^[19]^ Recent studies showed that GLP-1-based therapies may be effective and safe as an additional treatment to basal insulin in the peri-operative and critical care settings.^[20]^ GLP-1 RA, dipeptidyl peptidase 4 inhibitors, and metformin in the absence of acute kidney injury could as well take place in the treatment of COVID-19 patients with diabetes.^[20]^ Sodium-glucose transport protein (SGLT-2) inhibitors could take place in treating patients with diabetes and/or heart disease during hospitalization.^[21]^ In our study, we compared the effects of the usual basal-bolus insulin regimen versus combined alternative treatment modalities with or without insulin, on glucose control in COVID-19 infected patients, in a hospital setting.

## 2. Methods

This retrospective study used electronic medical records of type 2 diabetes patients hospitalized with COVID-19 diagnosis between April 1, 2020, and March 31, 2021. This study was approved by the Emek Medical Center Institutional Review Board (trial number 0029-21-EMC). Patients were included in the analysis if they were hospitalized with a diagnosis of COVID-19, had a preadmission diagnosis of type 2 diabetes, and were 18 years of age or older. Exclusion criteria were pregnancy, type 1 diabetes, diabetic ketoacidosis, or hyperglycemic hyperosmolar state.

The baseline characteristics included gender, age, body mass index, glomerular filtration rate, preadmission diabetes treatment regimens, and common diabetes-related comorbidities that is, cardiovascular disease, chronic kidney disease, and congestive heart failure.

### 2.1. Diabetes treatment

We divided the patients into 2 groups according to the diabetes treatment during hospitalization. The first group included patients treated with insulin only, and the second one was treated with other glucose-lowering medication with or without insulin.

Preadmission diabetes treatment was retrieved and classified into 4 groups as follows: a combination of long-acting and short-acting insulin, a combination of various oral antihyperglycemic drugs and insulin, oral antihyperglycemic medicine only, and diverse combinations of oral antihyperglycemic, GLP-1 agonist with or without insulin.

The most recent hemoglobin A1c (HbA1c) for each patient (within 6 months) before and after hospitalization, was retrieved. Average blood glucose during hospitalization was retrieved from the electronic charts of patients.

We explored the relationship between the multiple factors related to diabetes control during hospitalization through predictive ordinal regression analysis for categorical variables. Well-control patients were defined as having average blood glucose < 180 mg/dL, intermediate having average blood glucose of 180 to 240 mg/dL, and fair-controlled patients >240 mg/dL. The parameters included in the multivariant analysis were age, female or male sex, length of hospitalization, comorbidities (congestive heart failure, atherosclerosis, chronic kidney disease), insulin treatment, metformin treatment, SGLT-2 treatment, dipeptidyl peptidase IV, GLP-1 and sulfonylurea drugs, treatment with dexamethasone during hospitalization. Length of stay was categorized into 3 ranges: up to 3 days, up to 1 week, and >1 week of hospitalization.

### 2.2. COVID-19

COVID-19 severity was determined according to National Institute of Health criteria:

1. Critical disease: mechanically ventilated/multiorgan failure.
2. Severe disease: breath rate > 30, oxygen saturation < 93 while breathing ambient air, PaO2/FiO2 < 300.
3. Moderate disease: COVID-19 pneumonia on chest imaging.
4. Mild disease: fever > 38 and symptoms of COVID-19.

### 2.3. Statistical analysis

Continuous variables were presented as mean ± standard deviation or median (interquartile). Categorical data were compared by using the *χ*^2^ test. The HbA1C level was compared for patients by groups using the *χ*^2^ test before and after using a *t* test for paired samples. In a descriptive table, frequency and relative frequency were presented by groups – a type of treatment (insulin only and other drugs ± insulin) and change of treatment (changed and not). Odds ratio related to mortality was presented for categorical variables in correlation to levels of categorial variables, then the exposure factor is mortality.

A *P* value of <.05 was considered statistically significant. Analyses were performed with SPSS software (version 24.0; IBM, Armonk, NY).

Ordinal regression analysis is used to predict diabetes control with a set of independent variables.

## 3. Results

A total of 359 patients met the inclusion criteria. Clinical data of the patients included are presented in Table 1. In comparison with patients in the insulin-only group, more patients in the group treated with different antihyperglycemic medications with or without insulin were treated at home with oral medications or GLP-1 agonists (29% vs 51% and 6% vs 15%, respectively; *P* < .0001) More patients in the insulin-only group received the same insulin regimen at home, while few patients in the other treatments ± insulin group had only insulin therapy at home (38% vs 4%, respectively; *P* < .0001). No difference in preadmission HbA1c levels and insulin doses was found in the 2 groups of patients (Table 1).

**Table 1:** Clinical and demographic characteristics of the study patients.

	Overall	Insulin only	Other treatments ± insulin	*P* value
	N = 359	N = 197	N = 162	
Age (yr)	69 ± 13 [67]	68 ± 13 [71]	66 ± 13 [67]	.15
Male sex	189 (52.2%)	114 (50.7%)	86 (53%)	.66
BMI (kg/m²)	31 ± 7 [29]	31 ± 7 [29]	31 ± 6 [30]	.5
GFR (mL/min)	86 ± 61[71]	77 ± 61[60]	100 ± 60 [89]	.017
HbA1c (%)	7.9 ± 1.8 [7.5]	8.1 ± 1.9 [7.6]	7.7 ± 1.6 [7.4]	.08
Cardiovascular disease	143 (40%)	87 (44%)	62 (35%)	.26
CHF	52 (14.5%)	33 (16.7%)	19(11.7%)	.38
Chronic kidney disease	84 (23.2%)	62 (31.5%)	22 (13.5%)	.00
Insulin doses at home (units)	40 ± 30 (32)	42 ± 34 (33)	36 ± 23 (31)	.2
Insulin doses during hospitalization (units)	41 ± 31 (34)	41 ± 34 (34)	41 ± 27 (32)	.8
Length of stay (d)	10.1 ± 11	10.4 ± 9.6	9.7 ± 12.6	.5

For continuous variables the mean ± standard deviation [median] is presented; for categorical variables n (%).

BMI = body mass index, CHF = congestive heart failure, GFR = glomerular filtration rate, HbA1c = hemoglobin A1c.

### 3.1. Diabetes treatment during hospitalization

During hospitalization most of the patients in the combined treatment group were treated with metformin on top of insulin treatment (131 out 162 patients), whereas 28 patients received SGLT-2 inhibitors, 12 patients were treated with dipeptidyl peptidase 4 inhibitors, 12 with GLP-1 RA, and 7 patients with sulfonylurea.

There was no difference in insulin doses between the 2 groups during hospitalization (41 ± 34 vs 41 ± 27; *P* = .8).

**Figure 1.**
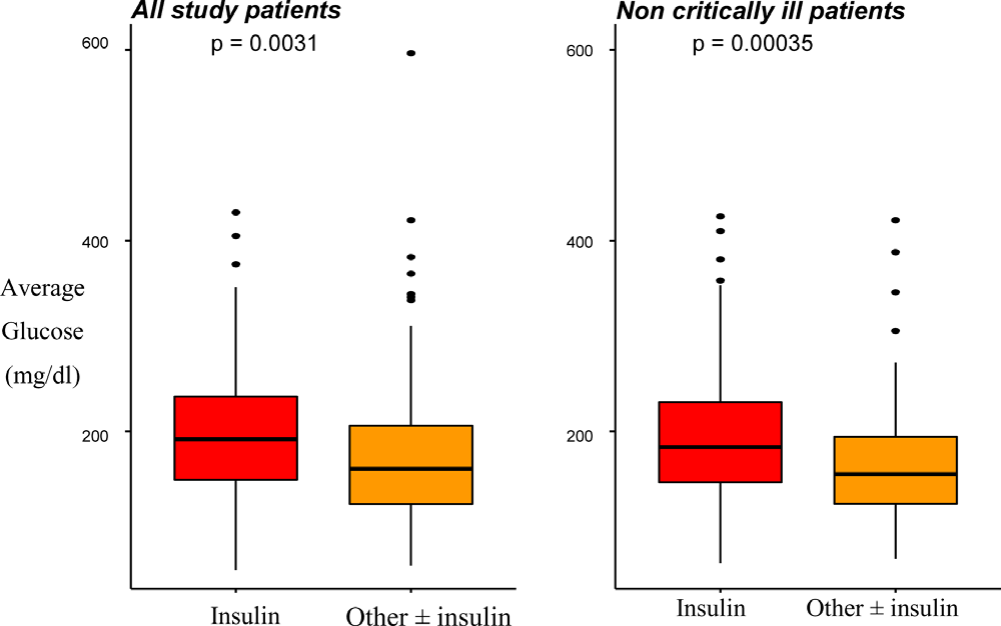
Average glucose level during hospitalization in 2 groups of treatment; before and after exclusion of mechanically ventilated patients.

Average blood glucose during hospitalization was higher in the insulin-only treatment group versus another treatment group (201 ± 66 mg/dL vs 180 ± 71 mg/dL, respectively; *P* = .004). After excluding mechanically ventilated patients, blood glucose remained significantly higher in the insulin-only group (192 ± 69 vs 169 ± 59 mg/dL, respectively; *P* = .003) (Fig. 1).

In patients with severe COVID-19, the average blood glucose was similar in the 2 study groups (211 ± 88 vs 225 ± 43 mg/dL in insulin-only and other treatment ± insulin groups; *P* = .3). However, in moderate severity COVID-19, the average blood glucose was significantly higher in the insulin-only treated group (197 ± 76 vs 168 ± 51 mg/dL; *P* = .001).

Ordinal logistic regression analysis which included multiple independent variables (age, female or male sex, comorbidities, length of stay, different antihyperglycemic treatments, kidney function, and dexamethasone treatment) revealed good correlations between antidiabetic therapy and average blood glucose (*R*² 30%, *P* = .02). The same model used for moderately ill COVID-19 patients was even more powerful (*R*² 40%, *P* = .002) (Table 2).

**Table 2:** Model of ordinal regression for prediction glucose control in moderately ill COVID-19 patients.

Model *P*	Goodness of fit			Pseudo *R*²	Variable	Test of parallel lines
	Pearson		Deviance	Negelkalke		
.002	0.15	0.915		0.41	Length of stay (*P* = .02)	0.002
					Age (*P* = .5)	
					Female or male sex (0.1)	
					Insulin (0.000)	
					Metformin (*P* = .12)	
					SGLT-2 i (*P* = .07)	
					DPP-IV (*P* = .1)	
					GLP-1a (0.2)	
					SFU (*P* = .5)	
					Comorbidities: 1 cardiovascular (*P* = .9)	
					2. CHF (*P* = .2)	
					3. CKD (*P* = .05) DEX (*P* = .6) eGFR e > 60 (*P* = .2) <60 (*P* = .6)	

CHF = congestive heart failure, CKD = chronic kidney disease, COVID-19 = coronavirus disease 2019, DEX = dexamethasone treatment, eGFR = estimated glomerular filtration rate, GLP-1a = glucagon-like peptide-1 agonists, SFU = sulfonylurea, SGLT-2 i = sodium-glucose transport protein inhibitor.

### 3.2. Survival

The overall survival in the 359 included patients was 65.5%. In the insulin-only treated group, 56.3% were alive at 28 days after admission, while in the other group 76% of patients survived (*P* = .2).

Patients who survived hospitalization had lower C-reactive protein levels at admission (11.5 ± 8.2 mg/L vs 16.1 ± 9.7; *P* = .000).

### 3.3. Diabetes control after discharge

In the 359 studied patients, HbA1c level decreased after hospitalization compared with preadmission value (7.5 ± 1.7 % vs 7.9 ± 1.8 %, respectively; *P* = .002). Also, in each treatment group diabetes control improved after hospitalization (Table 3).

**Table 3:** HbA1c level before and after hospitalization.

	Overall	Insulin-only group	Other treatments ± insulin	*P* value
	N = 359	N = 197	N = 162	
Before hospitalization	7.9 ± 1.8 (7.5)	8.1 ± 1.9 (7.6)	7.7 ± 1.6 (7.4)	.03*
After hospitalization	7.5 ± 1.7 (7.1)	7.6 ± 1.6 (7.2)	7.4 ± 1.8 (6.9)	.005*/.038

HbA1c = hemoglobin A1c.

* *P* value related to decreasing in HbA1c level after hospitalization in 2 treatment groups.

## 4. Discussion

Our study was conducted to assess glucose control in type 2 diabetes patients hospitalized with a diagnosis of COVID-19 infection. Diabetes has been reported as prevalent comorbidity in patients hospitalized with COVID-19 infection.^[22]^ Structured insulin regimens constitute the current recommendations for inpatient glycemic management in non-critically ill patients and remained the standard of care for uncontrolled hyperglycemia in patients hospitalized with COVID-19.^[23,24]^ In a recent real-life study in patients with moderate COVID-19 disease, treatment of hyperglycemia with continuous insulin infusion has been associated with reduced risk of severe disease and death,^[25]^ although it may be appropriate to continue preadmission non-insulin medications related to glucose control and associated metabolic conditions.^[23,24]^ In absence of prospective randomized trials, there is insufficient evidence to conclude if one of the specific classes of glucose-lowering agents is beneficial or harmful in people with COVID-19. Our study found that patients treated with different combinations of antihyperglycemic medications with or without insulin had better glucose control during hospitalization compared to patients treated with basal-bolus insulin protocol. The difference remains significant after the exclusion of mechanically ventilated patients from statistical analysis (*P* = .003). Interestingly, considering the COVID-19 severity, in patients with moderate severity disease, glycemic control was more adequate in the group treated with antidiabetic drugs with or without insulin compared to patients in the only-insulin-treated group (*P* = .008), whereas no difference between both groups of treatment was found in severely affected patients (*P* = .3). This finding corroborates the previous recommendations that insulin is still the most appropriate antihyperglycemic drug in critically ill patients with COVID-19 or uncontrolled hyperglycemia in non-critically ill patients hospitalized with COVID-19.^[25–28]^

Recent data showed that metformin, the first-line treatment for type 2 diabetes, was associated with significantly reduced mortality in patients with COVID-19.^[29,30]^ SGLT-2 treatment was not recommended according to some studies due to the risk of ketoacidosis and dehydration^[23,24]^ albeit in a recent study performed in hospitalized COVID-19 patients with and without diabetes, dapagliflozin treatment was well tolerated, and no side effects were reported.^[21]^

Remarkably, patients in the present study had overall better diabetes control before and after discharge compared to other studies that investigated glycemic control during hospitalization and after discharge.^[30,31]^ We assume that cohorts of patients with diabetes and COVID-19 are quite different from the typical diabetes patients admitted to the internal medicine department.

Some limitations must be acknowledged in the current analysis: the retrospective nature of the study; the decisions about the choice of treatment regimen during hospitalization may have been not homogeneous, partly at the discretion of the attending physician, partly given by an endocrinologist and a diabetes specialist nurse; the data was collected from only 1 center with consequently a relatively small sample size which may limit the extrapolation of the results. Nevertheless, some strengths should be highlighted: precisely the fact that the study was conducted in a single center may circumvent the relatively small sample size because of the standardized diabetes treatment protocols used in our center for more than a decade; mostly, the information about covariates like medications, comorbidities was available; in contrast to most similar studies which investigated the impact of drugs prior to admission with little data about the exposure to these medications during hospitalization, in our study we verified that the patients receive the medication during a hospital stay.

In conclusion, we conducted a retrospective real-life study to determine the impact of different diabetes treatment regimens on patients hospitalized with COVID-19. We found that mild to moderately affected patients could be safely treated with antihyperglycemic medications with or without additional basal insulin. In severely affected patients, basal-bolus insulin regimens remain the standard of care.

## Author contributions

**Conceptualization:** Elena Chertok Shacham.

**Data curation:** Elena Chertok Shacham, Nimra Maman, Avraham Ishay.

**Formal analysis:** Nimra Maman.

**Methodology:** Elena Chertok Shacham.

**Writing – original draft:** Elena Chertok Shacham.

**Writing – review & editing:** Avraham Ishay.
